# The role of imprinting genes’ loss of imprints in cancers and their clinical implications

**DOI:** 10.3389/fonc.2024.1365474

**Published:** 2024-05-15

**Authors:** Guojing Xie, Qin Si, Guangjie Zhang, Yu Fan, Qinghua Li, Ping Leng, Fengling Qiao, Simin Liang, Rong Yu, Yingshuang Wang

**Affiliations:** ^1^ Chongqing Key Laboratory of Sichuan-Chongqing Co-construction for Diagnosis and Treatment of Infectious Diseases Integrated Traditional Chinese and Western Medicine, College of Medical Technology, Chengdu University of Traditional Chinese Medicine, Chengdu, China; ^2^ Department of Clinical Laboratory, Chengdu Fifth People’s Hospital, Chengdu, China; ^3^ Sichuan Key Laboratory of Medical Molecular Testing, Chengdu, China

**Keywords:** cancer, diagnosis, progression, prognosis, epigenetic control, neoplastic gene regulation, gene imprint, methods

## Abstract

Genomic imprinting plays an important role in the growth and development of mammals. When the original imprint status of these genes is lost, known as loss of imprinting (LOI), it may affect growth, neurocognitive development, metabolism, and even tumor susceptibility. The LOI of imprint genes has gradually been found not only as an early event in tumorigenesis, but also to be involved in progression. More than 120 imprinted genes had been identified in humans. In this review, we summarized the most studied LOI of two gene clusters and 13 single genes in cancers. We focused on the roles they played, that is, as growth suppressors and anti-apoptosis agents, sustaining proliferative signaling or inducing angiogenesis; the molecular pathways they regulated; and especially their clinical significance. It is notable that 12 combined forms of multi-genes’ LOI, 3 of which have already been used as diagnostic models, achieved good sensitivity, specificity, and accuracy. In addition, the methods used for LOI detection in existing research are classified into detection of biallelic expression (BAE), differentially methylated regions (DMRs), methylation, and single-nucleotide polymorphisms (SNPs). These all indicated that the detection of imprinting genes’ LOI has potential clinical significance in cancer diagnosis, treatment, and prognosis.

## 1 Introduction

Genomic imprinting has significant roles in individual growth, development, and cell differentiation in mammals (1). In this epigenetic process, a small group of genes, called imprinted genes, are expressed depending on their parental origin. Imprinting is manifested mainly as silencing of transcription when a gene is expressed by one parent and activation of transcription when it is expressed by the other parent (2). When the original imprint status of imprinted genes is lost, known as loss of imprinting (LOI), silenced alleles are abnormally activated, or active genes are suppressed. Such imprint disorders can affect growth, neurocognitive development, metabolism, and even tumor susceptibility.

Insulin-like growth factor 2 (*IGF2*) is among the most studied genes affected by LOI in cancers. The LOI of *IGF2* gene was firstly demonstrated in wilms’ tumor (WT), a renal malignancy of childhood with an embryonic origin (3). LOI of the *IGF2* has also been found in some adult somatic tumors including colorectal cancer (CRC), renal cell carcinoma (RCC), stomach adenocarcinoma (STAD), and esophageal squamous cell carcinoma (ESCC) (4–7). The LOI of imprinted genes has gradually emerged as an early event in tumorigenesis, as well as being implicated in the development of tumors (8). Some studies have reported aberrant gene imprinting status in specific cancer types, whereas others have focused on the impact of these changes on tumors.

**Figure 1:**
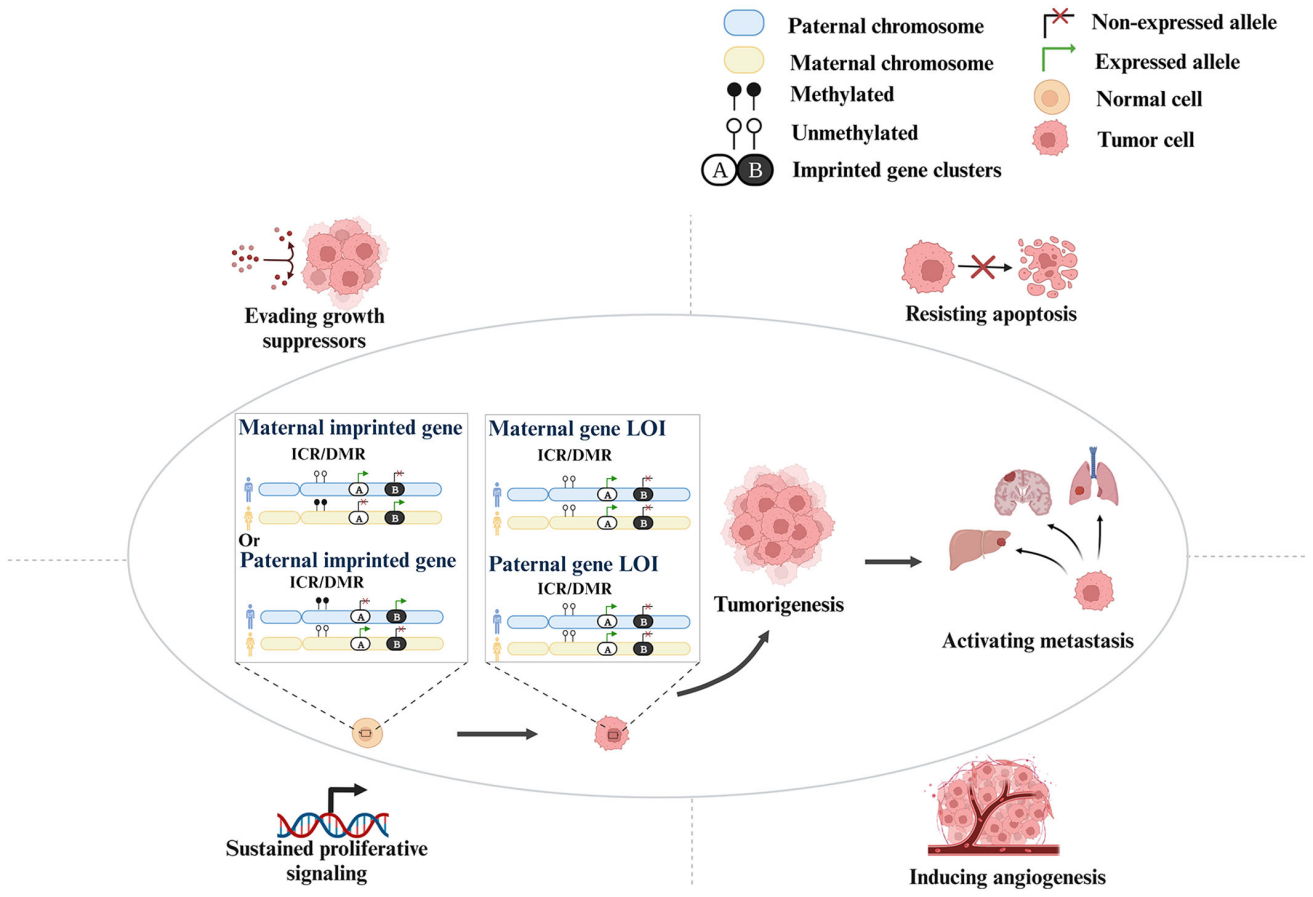
The molecular mechanism of LOI affecting cancers.

LOI affects tumorigenesis and progression mainly through conferring resistance to apoptosis and evasion of growth suppressors, sustaining proliferative signaling, inducing angiogenesis, and activating metastasis (**Figure 1**). For example, *IGF2* overexpression caused by LOI leads to the activation of the AKT and extracellular-regulated kinase (ERK) pathways, which promotes tumorigenesis (including cell proliferation and resistance to apoptosis) and metastasis (mainly liver metastases in CRC) (9, 10). Moreover, higher serum *IGF2* concentration is associated with metastasis in CRC, and is an indicator of poor prognosis (9). In triple-negative breast cancer (TNBC), the LOI of potassium two-pore domain channel subfamily K member 9 (*KCNK9*) gene involving differentially methylated region (DMR) hypomethylation leads to overexpression of the gene, increasing mitochondrial membrane potential and anti-apoptotic effect (11, 12). In human hepatocellular carcinoma (HCC), hypomethylation at CpG85 has been reported to lead to an increase in levels of an alternative RB1-E2B transcript and concomitant downregulation of the RB1 main transcript in confirmed retinoblastoma (*Rb*) LOI, resulting in the absence of the *Rb* pathway and the loss of its suppressor function (13). Inhibition of transforming growth factor-β (TGF-β) signaling increases the probability of malignancy (14). Hypermethylation of the DIRAS family GTPase 3 (*DIRAS3*) CpG has been found to lead to LOI, resulting in a decrease in its expression, blunting the Ras or phosphatidyl-inositol-3 Kinase (PI3K) pathway (15, 16). Defects in these feedback mechanisms could enhance proliferative signaling (17). The LOI of maternally expressed 3 (*MEG3*) inactivates its expression, thereby enhancing angiogenesis and promoting tumorigenesis (18, 19). These findings suggest potential actionable targets for LOI genes in cancers.

Disruption of the imprinting status also has implications for cancer diagnosis and prognosis. Studies have established diagnostic models using multiple imprinted genes based on the differences in allelic expression between normal, benign tumor, and cancerous tissues and have shown that these can function as efficient epigenetic biomarkers (20–22). Moreover, the presence of LOI at the delta-like non-canonical notch ligand 1 (*DLK1*) and *MEG3* locus has been found to vary between two different histological subtypes of rhabdomyosarcoma (RMS) (23). Therefore, LOI detection represents a novel tool for cancer diagnosis. For instance, patients with esophageal adenocarcinoma (EAC) <65 years old with *IGF2* LOI were found to have longer 5-year disease-free survival (DFS) (24), whereas in patients with CRC, the LOI was associated with higher overall mortality (4). These results demonstrate the importance of understanding the role of LOI in cancers and also illustrate the complexity arising from cancer tissue specificity.

More than 120 imprinted genes have been identified in humans (as displayed at geneimprint, http://www.geneimprint.com). However, there has been a lack of studies summarizing which imprinted genes are associated with cancers. Therefore, in this review, we used a systematic literature search strategy (**Supplementary Figure 1A**) to identify a total of 297 studies (after elimination of duplicate records). The two authors cross-checked the remaining articles, resulting in a total of 105 articles to be included in the review (the process is summarized in **Supplementary Data 1**, search strategy and selection criteria). These articles comprised results for 13 single genes (including two gene clusters) in 26 types of cancers and 12 combined forms of multi-gene LOI testing in 16 imprinted genes. Thus, our review provides a basis and prospective reference for the co-detection of imprinted genes and the selection of suitable biomarkers to establish novel clinical models in the future.

Imprinted genes are regulated by imprinted cluster-associated DMRs that play a critical part in maintaining parent-specific gene expression patterns known as imprinted control regions (ICRs). LOI is due to aberrant methylation in the DMRs of imprinted genes, usually loss of methylation maintenance, which produces aberrant transcripts that lead to activation of normally silent alleles. Methods for the detection of LOI have been established based on these mechanisms, such as the detection of biallelic expression (BAE), detection of DMR methylation, and detection of single-nucleotide polymorphisms (SNPs). In addition, 17 LOI detection methods are also summarized and grouped into three categories according to their principles, which will be helpful for selecting the appropriate LOI detection method in cancers.

## 2 Imprinted genes’ loss of imprint in cancers

LOI often occurs in many imprinted genes in malignancies, involving either a single imprinted gene LOI in one type of cancer or a specific cancer with multiple imprinted genes’ LOI simultaneously. Single genes, especially oncogenes or proto-oncogenes, may undergo alternations in expression when LOI occurs, subsequently affecting their biological functions in specific cancer types. Importantly, some of these genes are regulated in clusters. The chromosomal locations and regions that regulate imprinting and expression offer the potential for new therapeutic targets to be developed. **Table 1** provides a summary of LOI sites, expression levels, and clinical significance of 13 single imprinted genes in 26 types of cancers, as well as epigenetically mediated mechanisms of carcinogenesis, based on the literature. In addition, multi-gene testing has shown that most gene combinations are grouped in clusters or in similar positions; some of these combinations have already been used to establish tumor diagnostic models, showing impressive potential for direct clinical applications. These gene combinations could also provide an index for future diagnostic models. **Table 2** lists 12 gene combinations identified by multi-gene LOI testing, of which 3 have been established as cancer diagnostic models.

**Table 1:** Single-gene LOI in cancers.

Genes	Expressed alleles	Cancers	Form of LOIs/allelic switching or CNAs	Carcinogenic mechanism (regulators/signaling pathways)	Samples	Clinical parameters	Refs
DIRAS3, 1p31 AS	Paternal	GBMLGG	BAE^i^, CpG I or III hypermethylation	_	Tissues	Relevance to a longer overall survival.	(15)
DLK1, 14q32.2	Paternal	AML	BAE, region D1 (18 kb upstream of DLK1) hypermethylation	_	Blood, cell*(K562)	_	(25)
		EC	BAE, ICR, and MEG3 DMR hypermethylation	ΔDLK1: ↓proliferation, ↓tumorigenicity	Blood, cell^#^(NTera-2)	_	(26)
H19, 11p15.5 AS	Maternal	MMMT	BAE	_	Tissues	_	(27)
		EC	BAE	_	Tissue	_	(28)
		BLCA	BAE, ICR hypomethylation	_	Tissues	_	(29)
		ATL	BAE	_	Blood, cells^&^(KK1, SO4 and ST1)	_	(30)
		HNSC	BAE	_	Tissue, blood	_	(31)
HM13, 20q11.21	Unknown	BRCA	BAE, HM13 DMR hypomethylation/CNA	_	Tissues	_	(32)
IGF2, 11p15.5 AS	Paternal	HCC	BAE, ICR hypermethylation	_	Tissues	_	(33)
			BAE	_	Tissues	_	(34)
		PRAD	BAE	_	Tissues Normal tissues	_	(35)
			BAE, ICR hypermethylation	_	Tissues (no ICR hypermethylation) Normal tissues	_	(36)
		CRC	IGF2 DMR0 hypomethylation	_	Tissues	Relevance to higher overall mortality	(4)
			BAE, IGF2 DMR, and ICR hypomethylation	_	Tissues	_	(37)
			BAE	_	Blood	No relevance to smoking, alcohol, NSAIDs, and nutrient (calcium, folate, selenium, fiber, and fat).	(38)
			BAE	_	Blood	_	(39)
			BAE	_	Tissues, blood Normal tissues, blood	_	(40)
			BAE, IGF2 DMR, and ICR hypomethylation	_	Tissues, cells*(DKO-1, DKO-2, and DKO-3)	_	(41)
			BAE, ICR hypomethylation	_	Tissues Normal blood	No relevance to age, pathology stage, CEA value, or tumor size, respectively.	(42)
			BAE	_	Tissues Normal tissues	_	(43)
			BAE, ICR hypermethylation	_	Tissues Normal tissues	_	(44)
			IGF2 DMR hypomethylation	_	Tissues	No relevance to Dukes classification and pathologic status.	(45)
			BAE, IGF2 DMR hypermethylation	IGF2 LOI: ↑autophagy (CD133, p62, miRNA-195, IR-A, IGF1R, and GSK3β/PI3K-Akt-mTOR pathway)	Tissues, cells (Caco2^#^, HT-29^#^, HCT-8*, and HCT-116*), and normal cell (SW460*)	_	(46)
			BAE	_	Normal tissues	Relevance to a fivefold increased risk of adenoma formation.	(47)
		OS	BAE	_	Tissues	_	(48)
		WT	BAE, ICR hypermethylation	_	Tissues	_	(49–53)
			BAE, ICR hypermethylation	_	Tissues, blood Normal tissues	_	(54)
			BAE	_	Blood Normal tissues	_	(55)
			BAE	_	Tissues	_	(3, 56)
			BAE	_	Tissues Normal tissues	_	(57)
			BAE	_	Tissues	Relevance to greater diagnostic age (median = 65 months, IQR = 47–83 months) than normal imprinting (median = 24 months; IQR = 13–35 months).	(58)
			BAE, IGF2 DMRs hypomethylation	_	Tissues Normal tissues	_	(59)
		Insulinoma	BAE, IGF2 DMR2 hypermethylation	_	Tissues	Relevance to more advanced tumors but not to metastatic.	(60)
		EC	BAE, ICR hypermethylation	_	Tissues Normal tissues	Relevance to higher degree of lymph node involvement and metastasis but not to gender, age, cigarette, BMI, family history, depth of invasion, tumor differentiation, or stage.	(7)
			BAE, IGF2 DMR0 hypomethylation	_	Tissues	Relevance to a shorter survival time.	(61)
			BAE	_	Tissues Normal tissues	Relevance to a longer 5-year disease-free survival.	(24)
		LSCC	BAE	_	Tissues Normal tissues	_	(62)
		SFT	BAE		Tissues	_	(63)
		GBMLGG	BAE	_	Tissues	_	(64)
		OC	BAE	_	Tissues	_	(65)
		RCC	BAE	_	Tissues	Relevance to low-grade and low-stage tumors.	(5)
			BAE	_	Tissues	_	(66)
		RMS	BAE	_	Tissues	_	(67, 68)
		UCEC	BAE	_	Tissues	_	(69)
		BRCA	BAE	_	Tissues	_	(70, 71)
		ALL	BAE	_	Blood, bone marrow Normal blood	No relevance to recurrence rates, survival rates, and risk groups.	(72)
		AML	BAE	_	Blood, bone marrow	_	(73)
		STAD	BAE	_	Tissues Normal tissues, blood	Relevance to advanced stage tumors, without survival rates.	(6)
KCNK9, 8q24.3 AS	Maternal	BRCA	BAE, DMR hypomethylation	○KCNK9 DMR: ↑mitochondrial membrane potential, ↑anti-apoptosis (TASK3)	Tissues Normal tissues Cells (SUM225, HMEC15, MDA231, DKAT, SUM149, SUM190, and HEK293), normal cell (MCF10A)	No relevance to associate with age.	(11)
MEG3, 14q32	Maternal	NPC	MEG3 DMR (CpG45) hyper/hypomethylation/copy number loss (CpG45 hypermethylation)	↑MEG3: ↓proliferation (p53, p21, and MDM2/p53 pathway), ↓tumorigenicity	Tissue, cells (C666–1 and HK-1), normal cells (NP69, NP361, and NP460), and xenografts (xeno-666, xeno-2117, xeno-1915, xeno-99186, C15, and C17)	_	(74)
P57, 11p15.5 AS	Maternal	HNSC	BAE	_	Tissues	_	(75)
P73, 1p36.3	Maternal	STAD	BAE	_	Tissues	_	(76)
		EC	BAE/allelic switching	_	Tissues	_	(77)
		NA	BAE	_	Tissues	_	(78)
		RCC	BAE/allelic switching	_	Tissues	_	(79)
		BRCA	BAE	_	Tissues	_	(80)
PEG1, 7q32	Paternal	BRCA	BAE	_	Tissues	_	(81, 82)
		LC	BAE	_	Cells* (Ma10, HLC-1, RERF-LC-KJ, RERF-LC-AI, SQ-5, LC-1F, Ma2, Ma25, and LU65)	_	(83)
PEG3, 19q13.4 AS	Paternal	GBMLGG	BAE, promoter hypermethylation	ΔPEG3: ↑proliferation, ↓apoptosis (p53, β-catenin, and Siah1/Wnt pathway)	Tissues, cells (U87*, U343*, T98*, and D566)	_	(84)
	Paternal	GC	BAE	_	Cells* (JAR, 3A, JEG3, and BeWo)	_	(85)
Rb, 13q14.2	Maternal	HCC	BAE, DMR (CpG45) hypomethylation	_	Tissues, cells* (Huh7, HepG2, HLE, and HLF)	CpG85 hypermethylation is relevant to high overall survival (hyper: 34 weeks, normal/hypo: 156 weeks).	(13)
WT1-AS, 11p13	Paternal	WT	BAE, WT1 ARR DMR hypomethylation	_	Tissues	_	(86)

ALL, Acute lymphoblastic leukemia ; AML, Acute myeloid leukemia; ATL, Adult T-cell leukemia; BAE, Biallelic expression; BLCA, Bladder urothelial carcinoma; BRCA, Breast invasive carcinoma; CNAs, Copy-number aberrations; CRC, Colorectal cancer; EC, Embryonal carcinoma; GBMLGG, Glioma; GC, Gynecologic cancer; HCC, Hepatocellular carcinoma; HNSC, Head and neck squamous cell carcinoma; LC, Lung cancer; LSCC, Laryngeal squamous cell carcinoma; MMMT, Malignant mixed Müllerian tumor; NA, Neuroblastoma; NPC, Nasopharyngeal carcinoma; OC, Ovarian cancer; OS, Osteosarcoma; PRAD, Prostate adenocarcinoma; RCC, Renal cell carcinoma; RMS, Rhabdomyosarcoma; SFT, Solitary fibrous tumors; STAD, Stomach adenocarcinoma; UCEC, Uterine corpus endometrial carcinoma; WT, Wilms tumor. – (no reported or no cell experiments validated); i (inactivation); * (LOI’s exist); Δ (knock down); # (both LOI occurred and cell experiments validated); & (names of cells with LOI not indicated); ○ (demethylated); ↑ (upregulate or promote); ↓ (inhibit). Only samples’ LOI that occurred or experiments that were validated are counted; not all samples were used (unlabeled cells are only used for cell or animal experiments).

**Table 2:** Multiple-gene LOI in cancers.

Cancers	Location of imprinting clusters	Associated genes	Expressed alleles	Form of LOIs	Samples	Models	References
AML	11p13 AS	WT1/AWT1	Paternal	BAE	Cells (MOLT4, RS4:11, NALM20, and NB4)	A diagnostic tool to distinguish AML by AWT1 promotes hypermethylation.	(87)
	11p13	WT1-AS			Cells (NB4, KG1A, SKNO-1, and K562)		
BLAC	11p15.5 AS	IGF2	Paternal	BAE, IGF2 DMR hypomethylation	Tissues	_	(88)
		H19	Maternal	BAE, ICR hypomethylation	Tissues, normal tissues		
		IGF2	Paternal	BAE	Tissues	_	(89)
		H19	Maternal				
CC	11p15.5 AS	IGF2	Paternal	BAE	Tissues	_	(90)
		H19	Maternal				
CRC	11p15.5 AS	IGF2	Paternal	BAE	Tissues	_	(91)
	7q32	PEG1	Paternal				
	11p15.5 AS	IGF2	Paternal	BAE	Tissues, normal tissues	_	(92)
		H19	Maternal				
	11p15	LIT1	Paternal	BAE, KvDMR1 hypomethylation	Tissues		
GCT	11p15.5 AS	IGF2	Paternal	BAE	Tissues	_	(93)
		H19	Maternal				
	15q11.2	SNRPN	Paternal	5’ flanking region hypomethylation			
	11p15.5 AS	IGF2	Paternal	BAE	Tissues	_	(94–96)
		H19	Maternal				
		IGF2	Paternal	BAE, ICR hypomethylation	Tissues	_	(97)
		H19	Maternal		Tissues, normal tissues		
HCC	11p15.5 AS	IGF2	Paternal	BAE	Tissues, cells (HepG2, Hep3B, and Huh7)	_	(98)
		H19	Maternal		Tissues, cells (HepG2, Hep3B, Huh7, and PLC/PRF/5)		
		IGF2	Paternal	BAE	Tissues	_	(99)
		H19	Maternal				
		IGF2	Paternal	BAE, ICR hypermethylation	Tissues, normal tissues	_	(100)
		H19	Maternal	BAE, ICR hypomethylation			
	14q32	DLK1	Paternal	BAE, ICR hypomethylation	Tissues	_	(18)
		MEG3	Maternal				
HNSC	11p15.5 AS	IGF2	Paternal	BAE	Tissues	_	(101, 102)
		H19	Maternal				
LC	11p15.5 AS	IGF2	Paternal	BAE	Tissues	_	(103)
	7q32	PEG1					
	20q13.3	GNAS	Isoform dependent	BAE	Tissues	A diagnostic tool to distinguish LC by two or more positive genes of gene classes (GNAS, GRB10, SNRPN, and HM13).	(21)
	7p12-p11.2 AS	GRB10	Isoform dependent				
	15q11.2	SNRPN	Paternal				
	20q11.21	HM13	Unknown				
LUSC	11p15.5 AS	IGF2	Paternal	BAE, ICR hyper/hypomethylation	Tissues, normal tissues	_	(104)
		H19	Maternal	BAE, ICR hypomethylation	Tissues		
MB	11p15.5 AS	IGF2	Paternal	BAE	Tissues, normal tissues, and cell (MHH-MED-5)	_	(105)
		H19	Maternal		Tissues, cell (MHH-MED-2)		
Meningiomas	11p15.5 AS	IGF2	Paternal	BAE	Tissues	_	(106)
		H19	Maternal				
		MEG3	Maternal				
OC	11p15.5 AS	IGF2	Paternal	BAE	Tissues. HOC cells	_	(107)
		H19	Maternal	BAE, ICR hypermethylation	Tissues		
	11p15.5	KCNQ1	Maternal	BAE	Tissues. HOC cells		
	11p15	LIT1	Paternal	BAE			
	14q32	MEG3	Maternal	BAE			
	7q32	PEG1	Paternal	BAE, DMR hypermethylation			
	19q13.4 AS	PEG3	Paternal	BAE	Tissues		
	15q11.2-q12 AS	NDN	Paternal	BAE			
	11p15.5 AS	IGF2	Paternal	BAE	Tissues	_	(108)
		H19	Maternal				
OS	11p15.5 AS	IGF2	Paternal	BAE, ICR hypermethylation	Tissues	_	(109)
		H19	Maternal	BAE, ICR hypomethylation			
RMS	11p15.5 AS	IGF2	Paternal	BAE	Tissues	_	(110)
		H19	Maternal				
	14q32	DLK1	Paternal	BAE, ICR hypermethylation in ERMS	Tissues	A potential diagnostic tool to distinguish RMS subtypes by DLK1 and MEG3.	(23)
		MEG3	Maternal				
	11p15.5 AS	IGF2	Paternal	BAE, ICR hypermethylation			
		H19	Maternal				
Ten cancers	20q13.3	GNAS	Isoform dependent	BAE	Tissues	A diagnostic tool to distinguish 10 cancers by two or more positive genes of gene classes (GNAS, GRB10, and SNRPN).	(20)
	7p12-p11.2 AS	GRB10	Isoform dependent				
	15q11.2	SNRPN	Paternal				
WT	11p15.5 AS	IGF2	Paternal	BAE	Tissues	_	(111)
	11p15.5	IGF2AS	Paternal				
	11p13 AS	WT1/AWT1	Paternal	BAE, WT1 ARR DMR hypomethylation	Tissues	_	(112, 113)
	11p13	WT1-AS	Paternal				
	11p15.5 AS	IGF2	Paternal	BAE, ICR hypermethylation	Tissues	_	(114, 115)
		H19	Maternal				
	11p15.5 AS	IGF2	Paternal	BAE, ICR hypermethylation	Tissues	_	(116)
		H19	Maternal	BAE			
	11p15.5	KCNQ1	Maternal				
	11p15	LIT1	Paternal				
	11p15.5	TSSC5	Maternal				
	7p12-p11.2 AS	GRB10	Isoform dependent				
	14q32	MEG3	Maternal				
TC	15q11.2	SNRPN	Paternal	BAE	Tissues	A diagnostic tool to distinguish TC by two positive genes (SNRPN and HM13).	(22)
	20q11.21	HM13	Unknown				

AML, Acute myeloid leukemia; BAE, Biallelic expression; BLCA, Bladder urothelial carcinoma; CC, Cervical cancer; CNAs, Copy-number aberrations; CRC, Colorectal cancer; GCT, Germ cell tumor; HCC, Hepatocellular carcinoma; HNSC, Head and neck squamous cell carcinoma; LC, Lung cancer; LUSC, Laryngeal squamous cell carcinoma; MB, Medulloblastoma; OC, Ovarian cancer; OS, Osteosarcoma; RMS, Rhabdomyosarcoma; Ten cancer, Bladder, Breast, Colorectal, Esophagus, Gastric, Lung, Pancreatic, Prostate, Skin, and Thyroid cancer; WT, Wilms’ tumor; TC, Thyroid cancer. Only samples’ LOI that occurred are counted; not all samples were used.

### 2.1 Loss of imprint gene clusters

#### 2.1.1 IGF2-H19 locus

**Figure 2:**
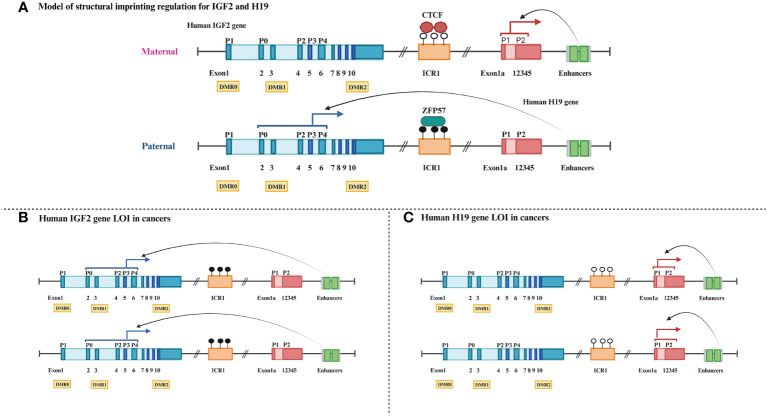
Schematic comparison of normal and loss of imprinting for human *IGF2-H19* gene cluster. **(A)** Dark blue boxes: *IGF2* exons, light blue boxes: *IGF2* introns, P0–P4: *IGF2* promoter regions, yellow rectangles: *IGF2* DMRs, orange rectangle: ICR1, black circle: methylated, white circle: unmethylated, red polygons: insulator binding protein CTCF, black green rectangle: transcription element *ZFP57*, dark red boxes: *H19* exon, light red box: *H19* introns, grayish green squares: cis-remote control element enhancers. **(B)** Blue solid arrows: parent-specific transcripts of IGF2. **(C)** Red solid arrows: parent-specific transcripts of H19.

*IGF2* and *H19*, located on chromosome 11p15.5 in humans, are a mutually imprinted pair of genes that share a common regulatory locus (**Figure 2A**) (117). The *IGF2* gene consists of 10 exons, and its expression is driven by five promoters (p0–p4) that possess different transcriptional activities both pre- and postnatally. In some cancer cells, four promoters (p0, p2, p3, and p4) whose *IGF2* mRNA transcripts are imprinted contribute significantly to total *IGF2* expression (118–120). Human *H19* expressing a long non-coding RNA (lncRNA) contains six exons and two promoters. *H19* DMR, also known as imprinting control region 1 (ICR1), is located between *IGF2* and *H19* and contains the binding sites for the epigenetic master regulator CTCF (121, 122). ICR1, *IGF2* promoter-specific DMRs 0, 1, and 2, which partially overlap the *IGF2* intronic and exonic sequences, the *IGF2* enhancer region downstream from *H19*, and imprinting factor zinc finger protein 57 (*ZFP57*) jointly play a crucial part in maintaining normal imprinting and expression of these two genes in mammals (123–125).

In most healthy adults, *IGF2*, which encodes proteins that promote fetal growth, is expressed only by the paternal allele (maternal ICR1 hypomethylation), whereas *H19*, which encodes an lncRNA with growth inhibitory properties, is expressed only by the maternal allele (paternal ICR1 hypermethylation) (126). This balance of expression of different parental alleles is broken when LOI occurs, routinely exhibiting opposing methylation states and biological functions, especially in the majority of patients with tumors. *IGF2* LOI associated with hypermethylation of ICR1 and hypomethylation of *IGF2* DMRs is prevalent and increases gene expression levels in the majority of cancers (**Figure 2B**) (4, 33). Moreover, ICR1 hypomethylation is also considered to be characteristic of *H19* LOI and regularly results in the upregulation of *H19* mRNA expression in human bladder cancer (**Figure 2C**) (29).

*IGF2* undergoes normal imprinting changes, can act synergistically with multiple signaling pathways, and participates in physiological processes (autophagy, oncogenesis, and glycemic metabolism) of patients. It is well known that IGF2/IGF1R binding exerts cellular autophagy mediated by inhibiting the PI3K-Akt-mTOR signaling pathway in the CRC (127). Activated glycogen synthase kinase-3β (*GSK3β*) can inhibit B-cell lymphoma-2 (*Bcl-2*) as a mediating event to stimulate autophagy (128, 129). A recent study demonstrated that *IGF2* LOI cancer stem cells (CSCs) were generally more prone to tumor formation and had higher levels of autophagy (CD133 with high expression and p62 with low expression) compared with maintenance of imprinting (MOI) cells in patients with CRC (46). Low expression of miRNA-195 in patients with CRC increased IGF2/IR-A binding, which more strongly promoted Akt expression and phosphorylation than IGF2/IGF1R, further decreasing GSK3β phosphorylation (46, 130). Overexpression of *IGF2* related to LOI and receptor tyrosine kinase genes including *DDR1, ERBB2*, and *FGFR1* have implicated the IGF2-INSR pathway in sphere formation of solitary fibrous tumor (SFT) (63). Hypoglycemia was also observed in SFT patients with *IGF2* LOI.

Several studies have assessed the clinical value of imprinted genes in tumors. LOI and ICR/DMR methylation and alterations in expression levels due to LOI are relevant to clinical parameters, especially those related to survival and mortality. LOI of *IGF2* was first identified in WT, which is a hereditary malignant embryonic tumor of infants (3), with a relatively older age at diagnosis of children with *IGF2* LOI (median = 65 months, IQR = 47–83 months) (58). However, subsequent studies also found *IGF2* LOI in adult somatic cell tumors, such as CRC, RCC, STAD, and ESCC. *IGF2* LOI has been reported to be associated with a fivefold increased risk of adenoma formation and higher overall mortality in CRC (4, 47). *IGF2* LOI appeared to predispose RCC patients to low-grade and low-stage tumors (5) and was more likely to occur in advanced STAD (6). Patients with ESCC with *IGF2* LOI showed a higher degree of lymph node involvement, metastasis, and shorter survival times (7, 61). However, patients with EAC with *IGF2* LOI were found to have a longer 5-year DFS (24). These not only show the importance of paying attention to LOI in cancers but also illustrate the complexity arising from cancer tissue specificity. Finally, *H19* LOI has been found to be present in patients with head and neck carcinoma, and patients with high expression of *H19* appeared to be more likely to experience relapse (31).

#### 2.1.2 Dlk1-MEG3 locus

**Figure 3:**
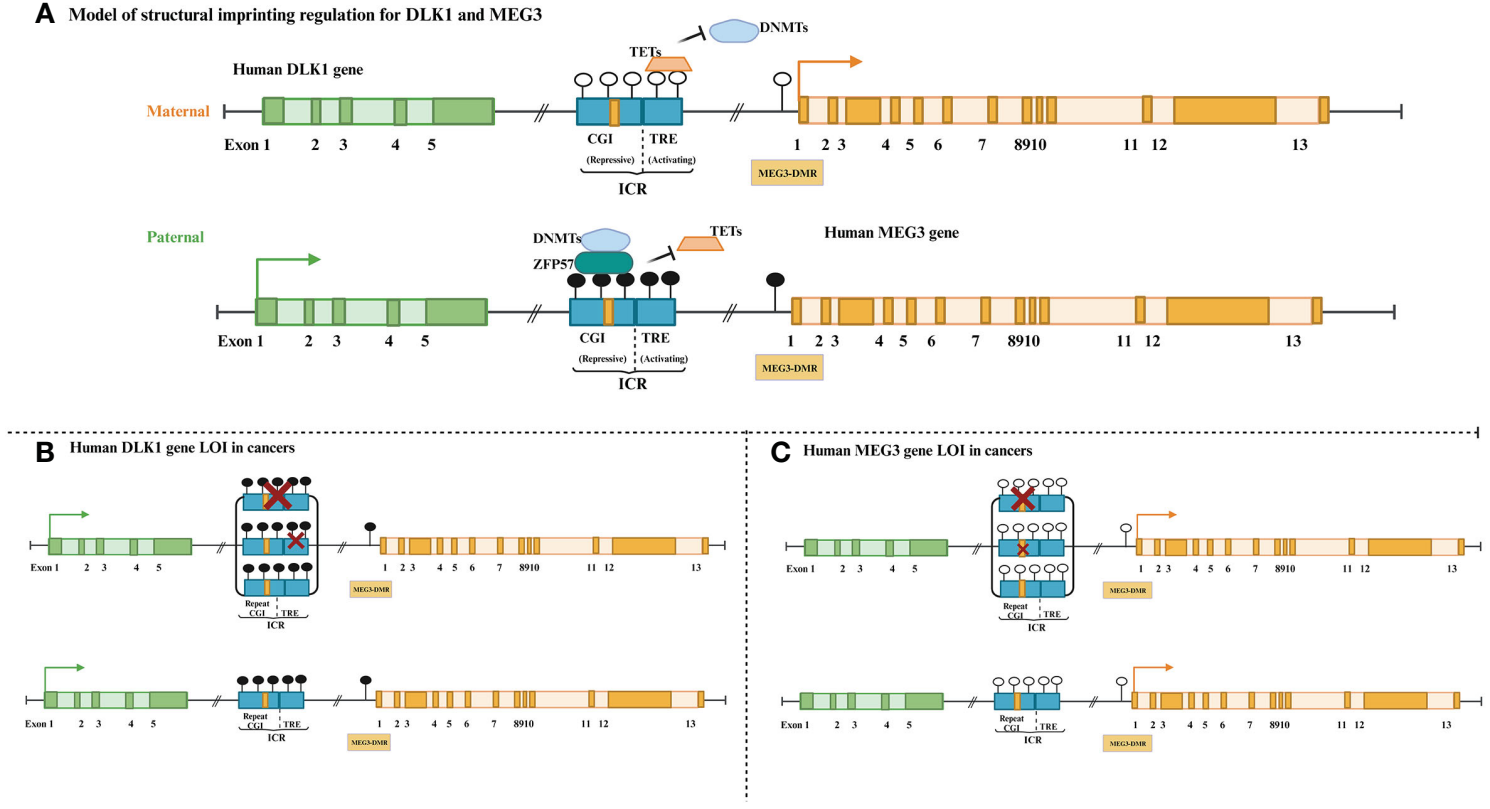
Schematic comparison of normal and loss of imprinting for human *DLK1-MEG3* gene cluster. **(A)** Dark green boxes: *DLK1* exons, light green boxes: *DLK1* introns, dark blue rectangles: ICR (CpG Island CGI and TRE work independently on different alleles to restrict the activities of TETs and DNMTs), yellow quads in CGI: conserved tandem repeat array, black circle: methylated, white circle: unmethylated, orange trapezoid: demethylated enzyme TETs, blue cloud: methylated enzyme DNMTs, black green rectangle: transcription element *ZFP57*, dark yellow boxes: *MEG3* exon, light yellow box: *MEG3* introns. **(B)** Green solid arrows: parent-specific transcripts of DLK1, red letter x: absence. **(C)** Yellow solid arrows: parent-specific transcripts of *MEG3*.

The human *DLK1* gene resides in the chromosomal 14q32 region, positioned with *MEG3*, with which it constructs an imprinted gene cluster (NCBI reference sequence: NC_000014.9). The paternally expressed protein-coding *DLK1* gene is composed of 5 exons, whereas *MEG3* with 13 exons maternally expresses an lncRNA. At the *DLK1-MEG3* locus, it is regulated by both the ICR and *MEG3* DMR containing the CTCF binding DNA sequence, which lies among the two genes (131, 132). Aronson et al. revealed that a hierarchical and unidirectional regulation existed between the ICR and *MEG3* DMR, and the dominant ICR was established as a dichotomous control element that maintained imprinting through allele-specific restriction of the DNA (de)methylation mechanism (**Figure 3A**) (133).

*DLK1* and *MEG3* are methylated on the paternal allele, but unmethylated on the maternal allele, which regulates their expression in healthy individuals (134). However, in some cancer patients, the parental alleles are expressed in an imbalanced manner and usually exhibit opposite methylation states and expression. *DLK1* LOI (ICR and *MEG3* DMR hypermethylation) manifests as biallelic *DLK1* expression and *MEG3* silencing, whereas *MEG3* LOI shows ICR and *MEG3* DMR hypomethylation and the opposite expression trend (**Figures 3B, C**). In addition to LOI, allelic switching (opposite single allele expression) accompanied by gains or losses of DNA methylation primarily on IG-DMR at the *DLK1-MEG3* locus had also been discovered in some patients with HCC (18). *MEG3* copy number loss was found only in patients with nasopharyngeal carcinoma (NPC) whose LOI manifested as DMR hypermethylation (74). These results indicate that genetics and epigenetics may synergistically influence the vast majority of tumors.

Similar to *IGF2* and *H19*, *DLK1* and *MEG3* also perform diverse biological functions in cancers. LOI was found to upregulate *DKL1* mRNA expression; however, knocking down its expression would inhibit proliferation and tumorigenicity in embryonal carcinoma (EC) (26). *DLK1* appears to exert a cancer-promoting role. Conversely, in glioma (GBMLGG), lower expression of *MEG3* promotes not only oncogenesis, but also malignant behavior such as proliferation, migration, and tumorigenicity (135). When restored to normal expression levels, *MEG3* played a tumor suppressor role suppressed by inducing a significant downward adjustment of focal adhesion kinase (FAK), vimentin, and inhibitory phosphorylation of non-receptor tyrosine kinase (SRC). Furthermore, MEG3 restoration increased levels of β-actin (an important skeletal protein), caveolin-1 (a negative growth regulator), and connexin-43, as well as activating N-myc downstream-regulated gene 1 (NDRG1), which has previously been shown to inhibit metastasis and migration in CRC (136). *MEG3* also increased expression of p53 and a potent cyclin-dependent kinase inhibitor called p21, which might explain the observed enhancement of G1/S cell cycle arrest, and stimulated E3 ubiquitin ligase MDM2 production, which could represent suppressed NPC metastasis through the p53-MDM2-Slug pathway (74).

With respect to clinical applications, LOI of imprinted genes combined may appear to be useful for differentiating tumor subtypes. It has also been shown that both embryonal and alveolar rhabdomyosarcomas (ERMS and ARMS, respectively) show LOI for the DMR of the *IGF2-H19* locus, while ERMS consistently shows LOI of the DMR at the *DLK1-MEG3* locus (23).

### 2.2 Single genes’ loss of imprint

#### 2.2.1 Rb, KCNK9, PEG3, and P73

Apart from the best-known genetic changes in the form of heredity, such as mutations, genomic instability, loss of heterozygosity (LOH) and copy number aberrations (CNAs) leading to the inactivation of oncogenes or proto-oncogenes, epigenetic change can also cause this phenomenon. In contrast to clustered genes, single-gene LOI exhibits BAE or dysregulation of aberrant transcripts. Alteration of an imprinting control center may lead to abnormal expression of oncogenes or tumor suppressor genes, causing different effects on promoting and suppressing cancer.

On the one hand, LOI genes that promote cancer comprise *Rb*, *KCNK9*, and paternally expressed gene 3 (*PEG3*). The *Rb* gene, a retinoblastoma susceptibility gene, was the first tumor suppressor gene to be cloned and have its full sequence determined. Anwar et al. identified that LOI (CpG85 hypomethylation) is also a novel pathway for the inactivation of *Rb* in HCC (13). The *Rb* gene expresses only the maternal gene, while the paternal gene expresses the abnormal transcript (RB1-E2B) that starts at the CpG85 island. In the absence of imprinting, levels of RB1-E2B will increase, eventually leading to decreased expression of the main transcript RB. Patients with CpG85 hypermethylation have shorter overall survival (the median survival rates for hypermethylation and normal/hypomethylation are 34 and 156 weeks, respectively). *KCNK9* LOI was found due to DMR hypomethylation, which leads to overexpression of its gene product, increasing mitochondrial membrane potential and anti-apoptosis in TNBC (11, 12). Hypermethylation of the *PEG3* promoter leads to LOI and decreased *PEG3* mRNA expression, increasing β-catenin levels, promoting proliferation, and inhibiting p53-dependent apoptosis in human GBMLGG (84). On the other hand, the LOI gene that inhibits cancer is *P73*. The increased expression of *P73*, including that resulting from LOI, could be a partial compensatory mechanism for defective p53 in ESCC (77).

### 2.3 Diagnostic models GNAS, GRB10, SNRPN, and HM13

Traditional cytology and histopathology, imaging examination, and use of serum biomarkers have contributed tremendously to the early detection of cancer, but accurate diagnostic assessment of nodules and early-stage cancers with insufficient evidence of tumor morphology or abnormal metabolism remains a great clinical challenge at present (137–140). However, epigenetics may compensate for this deficiency. There is already clear evidence that epigenetic changes during carcinogenesis often precede morphological changes (141, 142). To provide reference information for more accurate tumor-specific diagnosis and precise personalized treatment in clinical settings, we summarize 12 combined forms of multi-gene LOI testing in **Table 2**, of which 3 types of combinations have been established as cancer diagnostic models.

Some researchers have successfully exploited a novel method, quantitative chromogenic imprinted gene *in situ* hybridization (QCIGISH), targeting non-coding intron nascent RNA, to directly observe BAE, multiallelic expression (MAE), and total expression (TE) at transcription sites of imprinted genes in the nucleus to select these appropriate imprinted genes for the construction, optimization, and validation of tumor diagnostic models (20). First, a diagnostic model for 10 different solid cancer types (bladder, breast, colorectal, esophageal, gastric, lung, pancreatic, prostate, skin, and thyroid cancers) was built using imprinted genes’ GNAS complex locus (*GNAS*), growth factor receptor bound protein 10 (*GRB10*), and small nuclear ribonucleoprotein polypeptide N (*SNRPN*) with a total sensitivity of 94%, a specificity of 92%, and an accuracy of 93% (20). Next, based on the above preliminary model, a more specific diagnostic model for grading lung cancer (LC) was also established using *GNAS*, *GRB10*, *SNRPN*, and histocompatibility minor 13 (*HM13*). This diagnostic model was highly effective in the diagnosis of both different subtypes of LC and small lung nodules, with an overall sensitivity of 99.1%, a specificity of 92.1%, and an area under the curve (AUC) of 0.99 (21). Lastly, a thyroid cancer (TC) diagnostic model through imprinted genes *SNRPN* and *HM13* has achieved an overall diagnostic sensitivity of 100%, a specificity of 91.5%, a positive predictive value (PPV) of 96.5%, a negative predictive value (NPV) of 100%, and a diagnostic accuracy of 97.5% in a prospective validation (22).

In sum, these findings provide considerable benefits and ideas for screening or predicting appropriate tumor markers, comprehensive clinical risk assessment, and finding new epigenetic therapeutic targets. This fully reflects the importance and non-negligibility of tumor epigenetics.

## 3 Detecting methods of LOI genes in cancers

Various methods have been used in the detection of imprinted gene LOI in the past three decades. In the 105 studies listed in **Table 3**, restriction fragment length polymorphism PCR (RFLP-PCR) was the most frequently used method, used up to 84 times (75/105) from 1993 to 2020. This was followed by bisulfite sequencing PCR (BSP) (25/105, 2003–2021) and pyrosequencing (8/105, 2007–2014). LOI arises from abnormal methylation of the DMR of imprinted genes (usually loss of methylation maintenance), which produces double alleles (aberrant transcripts leading to silencing of a normally active allele). LOI can also be discriminated based on SNPs. According to the detection objects used, the 17 methods for this purpose can be categorized into three types: (I) detection of BAE: hot-stop PCR, nest PCR, QCIGISH, RFLP-PCR, real-time quantitative reverse transcription PCR, reverse transcription PCR, and pyrosequencing; (II) detection of DMR methylation: BSP, bisulfite PCR-Luminex, combined bisulfite restriction analysis, Illumina 450 K arrays, pyrosequencing, methylation-specific PCR, NOMe-sequencing, RFLP-PCR, and the MassARRAY EpiTYPER; and (III) detection of SNPs: SNuPE assays, RNA sequencing (RNA-seq), and DNA sequencing. To make the results more credible and convincing, there is a growing trend towards the simultaneous use of multiple analytical methods with the same or different principles and away from the use of single or single-principle methods in some studies.

**Table 3:** Detection methods of LOI in cancers.

Methods	Test objects	Years	Samples	References
DNA sequencing	BAE	2006	Tissues	(65)
Hot-stop PCR	BAE	2004	Blood	(38)
Nest PCR	BAE	2000	Tissues	(75)
QCIGISH	BAE	2020–2023	Tissues	(20–22)
RFLP-PCR	BAE	1993–2011	Tissues, cells, blood, and bone marrow	(3, 5, 6, 24, 27, 28, 30, 34, 35, 40, 55–58, 62, 64, 66–73, 76–83, 85, 89–91, 93–95, 98, 99, 102–106, 108, 110, 111)
RFLP-PCR and RT-qPCR	BAE	2006	Tissues, blood	(96)
RT-qPCR	BAE	2004–2021	Tissues, blood, bone marrow, and cells	(47, 87)
RT-PCR	BAE	2010	Tissues	(63)
Pyrosequencing	BAE, DMR methylation	2009	Tissues	(60)
RT-PCR and BSP	BAE, DMR methylation	2010	Tissues	(37)
DNA sequencing and the MassARRAY EpiTYPER	BAE, DMR methylation	2010	Blood, cell	(25)
DNA sequencing and pyrosequencing	BAE, DMR methylation	2011	Tissues	(36)
Fluorescent SNuPE assays and BSP	BAE, DMR methylation	2001	Tissues	(44)
Hot-stop PCR, RFLP-PCR, and pyrosequencing	BAE, DMR methylation	2007	Tissues	(116)
Hot-stop PCR and BSP	BAE, DMR methylation	2002–2003	Tissues and blood	(39, 41)
RFLP-PCR	BAE, DMR methylation	1994–2005	Tissues and blood	(52, 53, 59, 113, 115)
RFLP-PCR and BSP	BAE, DMR methylation	2000–2020	Tissues, cells, and blood	(7, 29, 31, 42, 46, 48, 49, 86, 92, 100, 112)
RFLP-PCR and COBRA	BAE, DMR methylation	2006–2008	Tissues and blood	(50, 51)
RFLP-PCR, COBRA, and BSP	BAE, DMR methylation	2008	Tissues	(15)
RFLP-PCR and isotope-labeled SNuPE assay	BAE, DMR methylation	1997	Tissues	(54)
RFLP-PCR and pyrosequencing	BAE, DMR methylation	2007–2014	Tissues	(61, 88)
RFLP and BPL	BAE, DMR methylation	2012	Tissues and cells	(107)
RNA-seq and Illumina 450 K array	BAE, DMR methylation	2018	Tissues	(32)
RT-PCR, MSP, COBRA, and BSP	BAE, DMR methylation	2003	Tissues	(109)
RT-PCR and BSP	BAE, DMR methylation	2006	Tissues and blood	(97)
RT-qPCR, BSP, and pyrosequencing	BAE, DMR methylation	2014	Tissues and cells	(13)
RT-qPCR, COBRA, and BSP	BAE, DMR methylation	2018	Blood and cells	(26)
RT-qPCR and COBRA	BAE, DMR methylation	2008	Tissues	(33)
RT-qPCR, DNA sequencing, BSP, and NOMe-Sequencing	BAE, DMR methylation	2021	Blood and cells	(11)
RT-qPCR, MSP, COBRA, and BSP	BAE, DMR methylation	2014	Tissues	(23)
BSP and pyrosequencing	BAE, DMR methylation	2012	Tissues and cells	(18)
BSP	DMR methylation	2008	Tissues	(45)
MSP	DMR methylation	2010	Tissues and cells	(84)
MSP and BSP	DMR methylation	2017	Tissues and cells	(74)
Pyrosequencing	DMR methylation	2010	Tissues	(4)
RFLP-PCR	DMR methylation	2011	Tissues	(114)

BAE, Biallelic expression; BSP, Bisulfite sequencing PCR; BPL, Bisulfite PCR-Luminex; COBRA, Combined bisulfite restriction analysis; MSP, Methylation-specific PCR; QCIGISH, Quantitative chromogenic imprinted gene in situ hybridization; RFLP-PCR, Restriction fragment length polymorphism PCR; RT-qPCR, Real-time quantitative reverse transcription PCR; RT-PCR, Reverse transcription PCR. Samples did not differentiate sources (normal controls or cancers).

Sequencing techniques based on sulfite treatment are widely used; however, despite their convenience, their drawbacks are also increasingly obvious. Sulfite treatment may lead to severe degradation of the input DNA owing to harsh reaction conditions, which is a common problem with most sequencing methods. Chemical enzymes compensate for this defect (143). For instance, a combination of chemical enzymes such as APOBEC3A (A3A) or engineered APOBEC3C (eA3C) and sequencing technologies has achieved consistent and reliable results (144, 145). This highlights the potential of multidisciplinary combinations to lead to new approaches.

Notably, several high-throughput techniques are being used for genomic methylation and allele-specific expression (ASE), showing great promise for the analysis and detection of imprinted gene LOI in cancers. The demand for comprehensive descriptions of DNA methylation patterns has led to a diversity of DNA methylation profiling technologies, including reduced representation bisulfite sequencing (RRBS) based on bisulfite conversion, methylated DNA binding domain sequencing, methylated DNA immunoprecipitation sequencing (MeDIP-seq) based on affinity enrichment, and methylation-sensitive restriction enzyme sequencing (MRE-seq) based on endonuclease digestion that targets genomic distribution (146). Recent studies have shown that utilizing the complementary properties of MeDIP-seq and MRE-seq can provide a rapid comparative analysis of the entire methylome at a fraction of the cost of whole-genome bisulfite sequencing (WGBS) (the gold standard method for detecting methylation at single-base resolution) with higher accuracy and reproducibility than either individual method (147–149). Analysis of existing RNA-seq datasets can be used to identify ASE of imprinted genes beyond evaluation of gene expression, thereby detecting the LOI of imprinted genes (150). However, when heterogeneous populations of cells, such as cancer samples, are analyzed, only single-cell measurements allowed the detection of widespread LOI events (151). Therefore, the use of effective and appropriate data analysis methods to analyze single-cell transcriptomic data will provide a major advantage in the analysis of tumor epigenetic aberrations. For example, BrewerIX, a standardized approach for the analysis of known imprinted genes, can be used to analyze RNA-seq data from single breast cancer cells to identify LOI of imprinted genes (151). Differential allelic expression using single-cell data (DAESC), a powerful method for differential ASE analysis using single-cell RNA sequencing (scRNA-seq) from multiple individuals, is capable of analyzing genes with differential ASE in pancreatic endocrine cells from patients with type 2 diabetes and controls, taking into account the effect of allelic switching, although it is not suitable for estimating cancer cells (152). These findings suggest that establishing standardized data analysis methods and combining existing LOI methods or potential methods with different characteristics may be a viable option in the cancer field, compared with exploring new detection methods that may have unknown limitations.

In conclusion, the presence or absence of LOI in cancers can be determined by using multiple methods of the same type vertically, two or more different types of methods horizontally, or even methods that combine multiple disciplines, making the results more accurate and reliable. Furthermore, the establishment of standardized data analysis methods for high-throughput technologies, in addition to combining multiple approaches, will help to uncover more potential imprinted genes and LOI, thereby facilitating the discovery of context-specific regulatory effects in cancers. As sequencing costs decrease, these methods will also be appealing in clinical practice.

## 4 Discussion

The established association between LOI and microsatellite instability (MSI) seems to provide a new epigenetic view of cancer susceptibility (40, 91), although this is complex, given the expression of imprinted genes in a parent-of-origin-specific manner. For the imprinted gene *Rb*, allele mutations from different parents have different effects on tumor susceptibility in hereditary retinoblastoma: if the mutation is of paternal origin, the offspring has a 12% chance of developing retinoblastoma, whereas when the mutation is of maternal origin, the offspring have a 75% chance of developing retinoblastoma (16, 153). Beyond embryonic-derived blastomas, epigenetic alterations in imprinted genes, often presenting as LOI, have been found in various somatic cancers. In addition, LOI of imprinted genes has been increasingly implicated in malignant behavior. The detection of LOI thus has potential clinical significance in cancer diagnosis, treatment, and prognosis.

Here, we have summarized 13 single-gene LOI in cancers, identifying the relevant detection sites and cancer types and considering whether they promote or inhibit functions in cancers. This provides a convenient index for co-detection of imprinted gene LOI in specific types of cancer. Moreover, as recent studies have found that aberrant gene imprinting patterns can occur together with cancer-associated CNAs (154) or allelic switching (77), we have also included these types of change in our analysis of studies (**Table 1**). Although the role of aberrant imprinting patterns in tumors is unquestionable, few studies have considered CNAs (1/70) or allelic switching (2/70) when reporting methylation profiles. Therefore, we suggest increasing the investigation of CNAs or allelic switching in future research to improve the accuracy of functional research on LOI genes. Coupled genes may be either clustered, as in the *IGF2-H19* locus or *DLK1-MEG3* locus, or non-clustered in specific cancers. In the analysis of loci for multi-gene detection panels, 12 combined forms of multi-genes were included, of which 3 gene combinations have been established as cancer diagnostic models. It is possible that more patterns may be found in the future based on the characteristics of imprinted genes in clustered LOI. There is also evidence to suggest that both the imprinting state and expression can be uncoupled in clustered genes. For instance, *IGF2* LOI was not found to be coupled with downregulation of *H19* expression in HCC (98, 99); in RMS, although *H19* LOI was present, the imprinting state of *IGF2* was maintained (110). These cases not only illustrate the complexity arising from cancer tissue specificity but also indicate an independent control mechanism for imprinting.

Notably, the role of imprinting gene LOI may vary among different tumors. For instance, the protein encoded by *p73* is structurally and functionally similar to that encoded by *p53*, a tumor suppressor. In *p53*-defect ESCC, *p73* was found to have elevated expression and LOI, which is speculated to be a substitute mechanism for the tumor-suppressing function (79). However, in RCC, LOI or switching of allelic expression of *p73* is associated with cancer development (77). On the other hand, even if a gene undergoes LOI, its downstream pathways may differ in different tumor types. In CRC, LOI of *IGF2* can enhance cell autophagy through the PI3K/Akt/mTOR pathway, whereas it might promote tumor formation through the IGF2-INSR pathway in SFT (46, 63). These findings suggest that it will be necessary for the future design of targeted LOI therapies to consider mutations of key factors in downstream pathways in different tumor types.

In the detection of imprinted genes’ LOI in cancers, although DNA methylation status changes are characteristic of LOI, their detection is distinct from that of overall DNA or promoter region methylation. Therefore, the focus should be on DMR/ICR only. In HCC, global loss of methylation and increased methylation at *DLK1* and *MEG3* DMR/ICR-specific sites have been simultaneously observed (18). Detection methods for LOI have evolved from qualitative to quantitative, from detecting overall CpG islands to single CpG site, and to more simplified procedures (**Supplementary Figure 1B**). Although we have summarized the mature LOI methods currently used in tumor detection based on the literature, when considering the depth of sequencing, sample requirements, and mutation detection, high-throughput methods such as whole-genome sequencing, whole-exon sequencing, and single-cell sequencing have great application prospects for LOI detection of imprinted genes in cancers (32, 155).

Both blood samples and tissue samples are suitable for the detection of LOI. *IGF2* LOI has been found in the blood and tissues of both patients with CRC and healthy controls and may be a valuable predictive marker of an individual’s risk of carcinoma (39, 40, 47). Although blood samples are more clinically accessible, tissue samples were more commonly used in the studies reviewed here (63/70 for single-gene detection, 34/35 for multiple-gene detection). This may be because in adult cancer patients, only the imprinted genes in cancer cells are LOI, while those in somatic cells maintain their imprint. With the development of enrichment methods for circulating tumor cells, use of tumor-derived exosomes in liquid biopsies, and advances in circulating cell-free DNA (cfDNA) methylation detection methods, blood samples have greater application prospects (156–158). Blood tests may therefore be of great informative value for large-scale LOI testing in cancer-susceptible populations.

## Author contributions

GX: Writing – original draft. QS: Writing – original draft. GZ: Writing – original draft. YF: Writing – original draft. QL: Writing – original draft. PL: Writing – original draft. FQ: Writing – original draft. SL: Writing – original draft. RY: Writing – original draft. YW: Writing – original draft.
