# Supplementary material for: The role of imprinting genes’ loss of imprints in cancers and their clinical implications
Source: Front Oncol. 2024 May 15;14:1365474. doi: 10.3389/fonc.2024.1365474 (PMC11133587; doi:10.3389/fonc.2024.1365474)
Supplement: Supplementary file 1 [file DataSheet_1.docx]

**Supplementary data 1 Search strategy and selection criteria**

*Search strategy*

The pieces of literatures involved in this study were searched from three **PubMed/Medline**, **Science Direct**, and **Web of Science** (last search updated on Dec 2023). The keywords used in the searching were "imprinting gene", "loss of imprinting" and "Neoplasm"/ "Cancer"/ "Tumor"/ "Tumour" not "review". To extend the search, related articles available in the database were examined, and all abstracts and citations were checked.

*Study selection—inclusion and exclusion criteria*

Two authors independently performed an initial selection of studies according to the titles and abstracts of all identified studies. A study was examined in detail if at least one of the authors selected it by title or abstract. Finally, a third author was consulted as to whether to include in the review questionable preselected studies.

Two authors screened these papers independently with the following these inclusion and exclusion criteria. Inclusion criteria: A. Studies examining imprinted genes, B. Human cancer related studies, C. Assays containing loss of imprinting of imprinted gene, D. Experimental original articles that provide data and evidence. Exclusion criteria: A. Studies not related to imprinted genes, B. No human cancer related studies, C. No mentioned or irrelevant to loss of imprinting of imprinted genes, D. Effect of Non-coding RNA on cancer.

**Supplementary figure legends**

**Supplementary figure. 1 A. Brief flow chart for literature searching and selection.** “N” refers to “number of studies”. **B. Methodological timeline and frequency rank charts.** It also describes time span and frequency rank of application of each method in the last three decades.
