# Supplementary figures and images for: The role of imprinting genes’ loss of imprints in cancers and their clinical implications
Source: Front Oncol. 2024 May 15;14:1365474. doi: 10.3389/fonc.2024.1365474 (PMC11133587; doi:10.3389/fonc.2024.1365474)

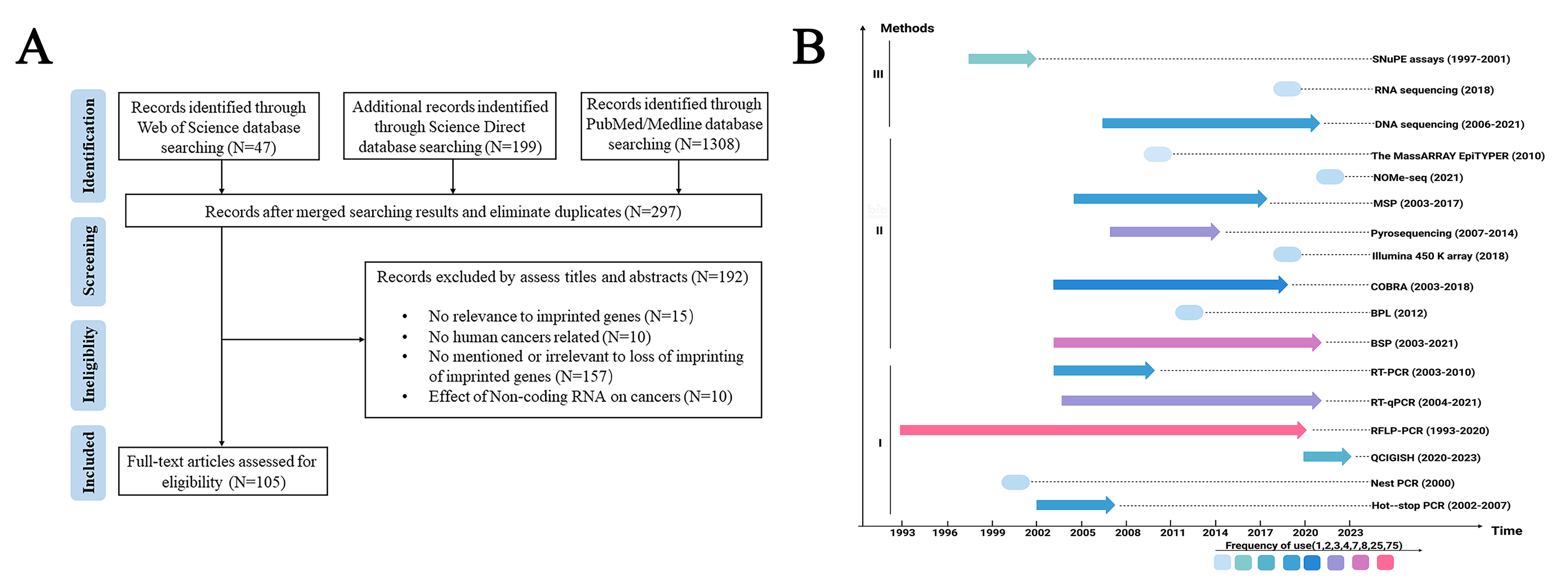

Supplement: Supplementary file 2 [file Image_1.tif]
